# Proteases: Importance, Immobilization Protocols, Potential of Activated Carbon as Support, and the Importance of Modifying Supports for Immobilization

**DOI:** 10.3390/biotech13020013

**Published:** 2024-04-30

**Authors:** Mateus Pereira Flores Santos, Evaldo Cardozo de Souza Junior, Carolina Villadóniga, Diego Vallés, Susana Castro-Sowinski, Renata Cristina Ferreira Bonomo, Cristiane Martins Veloso

**Affiliations:** 1 Programa de Pós-Graduação em Biologia e Biotecnologia de Microrganismos (PPGBBM), Universidade Estadual de Santa Cruz (UESC), Rodovia Jorge Amado, km 16, Ilhéus 45662-900, Bahia, Brazil; mateuspfloress@outlook.com; 2 Laboratório de Engenharia de Processos, Universidade Estadual do Sudoeste da Bahia (UESB), BR 415, km 04, s/n, Itapetinga 45700-000, Bahia, Brazil; evaldo.cardozo@uesb.edu.br (E.C.d.S.J.); crisvel@uesb.edu.br (C.M.V.); 3 Laboratório de Biocatalisadores e suas Aplicações, Instituto de Química Biológica, Faculdade de Ciências, Universidade da República, Iguá 4225, Montevideo 11400, Uruguay; cvilladoniga@gmail.com (C.V.); davc1972@gmail.com (D.V.); scs@fcien.edu.uy (S.C.-S.)

**Keywords:** carbonaceous support, derivatives, enzyme, functionalization, heterofunctional supports, immobilization methods

## 1. Introduction

Although enzymes have been used for thousands of years, their application in industrial processes has gained importance since the 20th century due to technological and scientific advances in several areas, including biochemistry. The enzyme market has become more attractive, with the large-scale commercialization and use of enzymes [1]. In this context, proteolytic enzymes stand out, being responsible for a large part of the financial movement in the sector, with applications in several areas. Obtaining proteases from different sources (plant, animal, and microbial sources) has provided a large variety of proteases with different properties (as specificity, stability, or activity). In addition, there is a greater range of applications of enzymes in academia and industry, attracting the attention of researchers focused on physiological and biotechnological applications [1,2].

Proteases stand out in food science and technology, with wide applications in the food industry. They are used in the preparation of protein hydrolysates, catalyzing the hydrolysis of peptide bonds in proteins. In the global market for proteases, several plant proteases have a predominant and almost exclusive role in specific applications then those from other sources being papain (from papaya latex), bromelain (from pineapple stems), and ficin (from the latex of the fig tree) most relevant [3].

Among the proteases from animal sources, pepsin and trypsin have been considered as the most efficient proteolytic enzymes, being released by the stomach and pancreas, respectively. They work together with chymotrypsin and carboxypeptidase in the metabolic processing of protein hydrolysis into easily absorbed essential peptides and amino acids. These amino acids have several functions in the body, such as muscle growth and hormone production [4,5]. 

Despite the high catalytic efficiency of these hydrolytic enzymes in their soluble form, factors such as high cost and low stability under some conditions have limited their use in industrial processes. Therefore, the use of these enzymes in their immobilized form has become a promising alternative since immobilized enzymes have several advantages including multiple uses (reuse), better process control, and mechanical stability when compared to the enzymes in solution, as well as the enhancement of the catalytic efficiency in some cases [6].

Enzymes can be immobilized by a variety of methods, which are based on chemical and/or physical binding and the reversibility of the binding process. Physical adsorption (physisorption) is a reversible process, characterized by weak interactions between the enzyme and the support. On the other hand, chemical adsorption (chemisorption) is considered an irreversible process with stronger interactions, with a greater amount of energy involved in the process. Among the chemical immobilization methods, the cross-linking [7] and covalent bonding stand out [8,9,10], while encapsulation and adsorption stand out as the physical methods [11,12]. 

The immobilization method should guarantee enzymatic activity and stability for long periods, in addition to preventing desorption, making it reusable and allowing for the free diffusion of substrates and reaction products [10]. The selection of the immobilization method should be based on various parameters such as the process efficiency, the costs of the immobilization procedure, the toxicity of the immobilization reagents, and the final properties required in the derivative [13]. In general, adsorption and covalent bonding are the most used techniques for enzyme immobilization [5]. 

The immobilization of biomolecules can be performed using numerous matrices, which are classified according to their origin as organic supports (natural and synthetic); inorganic supports (natural and synthetic); and hybrid supports (materials of organic and inorganic origin) [14,15]. Among the groups of supports, natural organic matrices are widely used due to their low cost of synthesis and application. However, inorganic substrates have stood out because they have intermediate production costs and are more suitable for industrial use due to their high mechanical resistance, good thermal stability, resistance to organic solvents, and resistance to attack by microorganisms [15]. 

In the group of materials of organic origin, activated carbon has been identified as a support for enzyme immobilization. In general, this material is used in adsorption processes due to its versatility of application and low production cost once it has been synthesized from agro-industrial residues. In addition, activated carbon has several important properties, such as a high surface area, good chemical, mechanical, and thermal stability, hydrophilicity, and insolubility, due to the presence of hydrophilic functional groups on its surface, as well as the ease of separation and reuse, which are important characteristics for use on an industrial scale [16]. Several studies have assessed the efficiency of activated carbon in different processes, including the immobilization of pancreatin on activated carbon [17], papain immobilization [18], and the immobilization of digestive enzymes on activated carbon to obtain bioactive hydrolysates [5,10].

As reported by Ramani et al. [19], activated carbon has characteristics that allow for surface modification to improve its properties for enzyme immobilization. Among the methods used for the synthesis of activated carbons with heterofunctional surfaces, the modification using glutaraldehyde has been widely used for enzyme immobilization due to its efficiency [11,19]. However, novel techniques for modifying the supports have been studied to replace glutaraldehyde, due to its toxicity [20]. The metallization of supports for enzyme immobilization has gained prominence, due to the low toxicity of the reagents and ease of execution. The ions interact with the amino acid residues present in the enzymes and, in some cases, the ions act as cofactors, improving the enzyme activity [21]. 

This review study will address immobilization methods with potential applications for protease immobilization. Special focus will be given to methods that use hydrophobic supports, such as activated carbon. The modification methods of the supports will also be discussed, with an emphasis on those aimed at enzyme immobilization on activated carbon. 

## 2. Proteases

Proteases (peptidases) are enzymes that catalyze the hydrolysis of peptide bonds in proteins or peptides, leading to the release of peptides of variable sizes [22]. These enzymes play important roles in food science and are used in various sectors of the food industry, including meat tenderization, beverage clarification, bakery, the production of high maltose syrup, flavor enhancement, waste treatment, and the preparation of protein hydrolysates, among others. Protein hydrolysates, for example, have properties that make them attractive as a source of amino acids for human nutrition, with better physiological behavior when compared to native proteins, once oligopeptides, mainly di- and tripeptides, are better absorbed by the body and have a better balance of amino acids when compared to free amino acids [23,24].

In the international enzyme classification (EC number) nomenclature, peptidases belong to class 3 and subclass 3.4, which is further divided into two groups: exopeptidases and endopeptidases. Exopeptidases catalyze the hydrolysis of peptide bonds at the N- or C-terminal end of polypeptide chains and are called aminopeptidases and carboxypeptidases, respectively. Endopeptidases act preferentially in the inner regions of the polypeptide chains [25].

Also, proteases can be classified according to the chemical nature of their catalytic site or their mechanism of action so that each class of proteases has a particular set of amino acids in its active site. EC use catalytic type for classification and nomenclature. They are originally classified into 4 groups: (i) serine proteases, which have the catalytic triad consisting of Ser, His, and Asp; (ii) cysteine proteases, which have the amino acids Cys, Asp, and His; (iii) endopeptidases or aspartic proteases, which have two Asp amino acids; and (iv) metalloproteinases or metalloproteases presenting a metal ion at the active site and more recently, the threonine group was included [25].

Rawlings and Barrett proposed a new system of classification according to peptidase molecular structure and homology. Amino acid sequencies and three-dimensional structures data available. The system’s beginnings were described in 1993 [26] and was accessible on the web as the MEROPS database (URL: https://www.ebi.ac.uk/merops, accessed on 25 March 2024) in 1996 [27].

The MEROPS database orders the data into three classification levels: Clans, Families, and Peptidases.

A clan contains peptidases that have evolved from a single ancestral protein. It is formed by one or more families that show evidence of their evolutionary relationship by similarity in three-dimensional structures, or when structures are not available, by the order of catalytic-site residues in the polypeptide chain and often by common sequence motifs around the catalytic residues. A family is composed of homologous peptidases, with statistically significant similarities in amino acid sequences to at least one member of the family, considering the part of polypeptide chain responsible for peptidase activity (termed the “peptidase unit”). Finally, each individual peptidase has a particular activity, structure, or genetics [27]. 

We will describe some characteristics of some relevant animal and plant proteases of natural origin. Among them, there are enzymes from the digestive system of mammals: pepsin and trypsin, and the enzymes of plant origin, papain and bromelain.

### 2.1. Pepsin

Pepsin (E.C. 3.4.23.1) is an aspartic endopeptidase belonging to the family A1 of the clan AA in the MEROPS database [28]. It presents a characteristic pair of catalytic aspartate residues, forming a catalytic dyad, and is also known as acid protease. It is found in the stomach, combined with chymotrypsin and trypsin, which are other proteolytic enzymes of the digestive system. During digestion, each of these enzymes cleaves particular peptide bonds, digesting dietary proteins into peptides and amino acids, which will be absorbed by the intestinal mucosa [29]. 

Pepsinogen, which is a zymogen (pro-enzyme), is the inactive form of pepsin initially released in the digestive process, which becomes active (pepsin) in contact with hydrochloric acid in the stomach. The pepsin activation occurs through the partial digestion of segments of the pepsinogen polypeptide chains. In addition to activating pepsin, hydrochloric acid is responsible for maintaining the pH of the medium in the acidic range, guaranteeing the activity of pepsin, which has no activity at neutral/basic pH values [30].

Pepsin has an approximate molecular mass of 35 kDa and an isoelectric point close to pH 1.0. As it is found in the digestive system, it acts at a more acidic pH, ranging from 2.0 to 4.0. In the food industry, it is used as a milk coagulant for cheese production and in the preparation of protein hydrolysates from plant and animal sources, which can be used as flavoring agents in food or beverages, as well as in the hydrolysis of soy allergens [1,10,23].

### 2.2. Trypsin

Trypsin (EC 3.4.21.4) is a serine endopeptidase, belonging to the S01 family of the subclan PA(S), clan PA in the MEROPS classification, which is characterized by the presence of a catalytic triad composed of histidine, aspartate, and serine. From the presence of these three amino acids forms a charge relay system that functions by the transfer of electrons from the carboxyl group of Asp to the oxygen of Ser, which then becomes a powerful nucleophile, capable of attacking the carbonyl carbon atom of the peptide bond of the substrate. The presence of metal cofactors such as Fe^2+^, Mg^2+^, Mn^2+^, and Zn^2+^ can enhance the trypsin activity, among other non-protein components [5,31].

Like pepsin, trypsin is a protease of the digestive system, produced by the pancreas in an inactive form called trypsinogen, becoming active in the small intestine through partial hydrolysis by enterokinase. It has a structure very similar to chymotrypsin, but differs in substrate specificity, preferring the cleavage of peptide bonds on the carboxyl side of lysine (Lys) or arginine (Arg), except when they are followed by a proline (Pro) [5,25].

It has an approximate molecular mass of 23 kDa and an isoelectric point close to pH 10.5. Trypsin is active at basic pH, in a range from 7.0 to 9.0, once it is an enzyme found in the digestive system that acts in the small intestine. In the food industry, it can be used as a milk coagulant for cheese production, as well as in the synthesis of food flavor hydrolysates (mainly those replaced by microbial proteinases) [1,31].

### 2.3. Papain

Papain (EC 3.4.22.2) is a plant cysteine protease isolated from papaya (*Carica papaya* L.) latex, belonging to the family C1 (subfamily C1A) of the clan CA (MEROPS) and is the founding member of the C1 family. It is single-chain globular protein of 212 amino acids with a molecular mass of 23,406 Da containing four disulfide bonds, and two domains separated by the active site cleft. The L- or N-terminal domain consists mainly of a set of α-helices, while the R- or C-terminal domain presents mostly antiparallel β-strands [32]. 

In the catalytically active enzyme, Cys 25 and His 159 are located in each domain at the bottom of the “V” shaped active site cleft on top of the molecule [33]. A long α helix from the N-terminus runs through the middle of the molecule, and the catalytic cysteine is found at the end of it. This enzyme, like other cysteine proteases, prefers an amino acid containing a large hydrophobic side chain at position P2 and does not accept Val at P1’ [34].

Papain is stable and active under a wide range of conditions, showing unusual stability to high concentrations of denaturing agents, such as 8 M urea or organic solvent like 70% EtOH. The optimum pH for activity of papain is 3.0–7.0, which varies with the different substrates, and it is very stable even at elevated temperatures [35].

Papain has multiple uses, ranging from meat softening [36], beer clarification, yeast extract production, and dental cleaning to more specific applications that require a purified preparation, as needed by applications in cosmetology and medicine [37].

### 2.4. Bromelains

The name bromelain is currently used to refer to the major cysteine endopeptidases of pineapple (*Ananas comosus* L.), belonging to the family C1 (subfamily C1A) of the clan CA (MEROPS). Stem bromelain and fruit bromelain are the enzymes responsible for the majority proteolytic activity of pineapple stem and fruit juices, respectively [38]. Additionally, two minor cysteine endopeptidases (ananain and comosain) were purified from the pineapple stem [39]. These enzymes require reducing agents, such as β-mercaptoethanol, to express its maximum activity.

Stem bromelain (EC 3.4.22.32) is the predominant protease (almost 90%) in Ananas comosus stem extracts. It is a single 24.5 kDa glycosylated polypeptide chain with an isoelectric point (pI) of 9.55 [40] and contains seven Cys residues and three disulfide bonds. The complete amino acid sequence has been deduced by Ritonja et al. [41], with a molecular mass of 22,831 Da. A wide optimum pH, both with synthetic and protein substrates, was reported. Despite its high activity on different protein substrates, it acts efficiently on synthetic substrates that contain Arg–Arg bonds. A particular preference of the enzyme for the hydrolysis of bonds between polar amino acids has also been reported. A distinctive feature of this protease is its relatively weak inhibition by E-64 and its lack of inhibition by chicken cystatin, which differentiates it from most C1 family peptidases [40].

This extract, called “fruit bromelain” (EC 3.4.22.33), constitutes 30–40% of the total protein from the Ananas cosmosus fruit pulp and almost 90% of the proteolytically active material. It is a single polypeptide chain of approximately 25 kDa whose pI is 4.6 [42]. The N-terminal sequence is identical to stem bromelain but is immunologically distinct from it and from ananain. Like stem bromelain, it has a broad optimum pH against protein and synthetic substrates. In addition, it prefers the synthetic substrate: Bz-Phe-Val-Arg-NHMec but it is not capable of hydrolyzing the preferred stem bromelain substrate—Z-Arg-Arg-NHMec—as a distinguishable feature [43].

Bromelain’s therapeutic potential is due to its biochemical and pharmacological properties. Bromelain is widely used in biotechnological and pharmaceutical applications such as meat tenderization, brewing, baking, and producing bioactive peptides from protein hydrolysis. Other multiple uses of these enzymes are tanning and hair removal in the leather industries, the textile and wool industries, and cosmetic and detergent formulations. In addition, Bromelain has also been used in folk medicine as a wound healer, anti-inflammatory, anti-diarrhea, and digestive aid [44,45].

### 2.5. Ficin

The latex of fig (*Ficus carica*) constitutes an important source of many cysteine proteases, known under the general term ficin or ficain (EC 3.4.22.3), which belongs to the papain family (family C1A, clan CA, MEROPS) [46]. The optimal pH range for ficin is from 5.0–8.0 and the optimum temperature is 45–55 °C. In studies of the 3D structure and amino acid sequences, the residues around the catalytic cysteine were found to resemble the corresponding sequence in papain for the neighboring residues of the active site [35]. Like papain, ficin has a significant resistance to denaturation by urea and ethanol [47].

Ficin is used as an exogenous enzyme for commercial meat tenderization. This enzyme, along with papain and bromelain, have been approved and are generally considered safe (GRAS) for use in the meat industry by the US Department of Agriculture [48]. Commercial preparations are used in the brewing industry to obtain good colloidal properties at low temperatures and to produce fish protein hydrolysate (FPH) among other applications [49].

## 3. Immobilization

Although enzymes have many advantages when compared to chemical catalysts, such as high catalytic activity, specificity for a particular substrate, and high activity in mild reaction conditions, their use is limited in some industrial processes due to thier high cost associated with higher purity, and its low stability under certain operating conditions. In addition, the difficulty of separating the enzyme from the final product impairs its use in continuous processes and large-scale applications. In contrast, the immobilized enzymes have been used for enzymatic catalysis and has overcome the deficiencies of this process [50].

Enzyme immobilization is a generic term used to describe the enzymes physically confined or localized in an inert support/matrix with the retention of their catalytic activities, which can be used repeatedly and continuously. In addition, the immobilization process can facilitate the separation of the enzyme from the final product and increase its stability by reducing the changes in the native structure by the environmental conditions (temperature, pH, and organic solvents), which is attractive for the application of enzymes in the industrial sector [51]. 

In 1916, the first scientific study on enzyme immobilization was carried out through invertase immobilization by adsorption on a carbon matrix. Later, this technique improved upon current immobilization techniques. Improvements of immobilization techniques were performed during the 1950s and 1960s, but in 1960, different immobilization methods by covalent bonds were developed, and their application in chemical processes has been studied to date, with more than 10,000 publications and patents filed about the different enzyme immobilization techniques [52,53].

According to Motevalizadeh et al. [54], from a commercial point of view, the main interest in immobilizing an enzyme is to obtain a biocatalyst with activity and stability that are not affected during the process when compared to its free form. The use of supports and/or techniques that prevent structural changes in the active site is required to avoid catalytic activity losses during its use. The advantages of immobilization include the reuse of enzymes, continuous operating processes (use of fixed bed or batch reactors without need of membrane to isolate enzyme from product), the easy separation of product-substrate, minimized enzyme losses by desorption, process repeatability, and high stability, among others.

Basso and Serban [55] state that enzymatic immobilization brings important industrial advantages such as the use of simplified and/or continuous processing. However, the option of using industrially immobilized enzyme depends on the economic evaluation of the costs associated with its immobilization and use versus benefits obtained in the process, comparing them with those of the soluble enzyme.

The use of immobilized enzymes in industry is increasing as they can be recovered and reused many times maintaining activity for long periods of time and are applicable to a variety of processes [56].

However, the immobilization procedure presents some disadvantages, such as the conformational changes occurring in the structure of the enzyme, depending on the immobilization procedure, leading to the enzyme immobilization in an inactive form and the possible loss of enzyme activity during immobilization. Another disadvantage are the diffusional effects caused by the low transport of the substrate and the product due to limitations in the access of the substrate to the active site of the enzyme. Therefore, the cost of immobilization, as well as the methods used, should be considered based on the biocatalyst’s shelf life. To minimize these disadvantages, knowledge about the nature of the enzyme, the material used as support for the immobilization, and the immobilization technique is required [57,58].

In general, the use of hydrophobic supports is advantageous for practical applications due to their convenience in handling, the ease of separation, greater stability, the possibility for reuse, and the prevention of interactions with interfaces, among others. Concerning the immobilization methods, they are based on the type of interactions between the support and the enzymes and can be classified as chemical methods or physical methods. Chemical methods require high binding energy such as covalent bonding, cross-linking, and affinity. Physical methods have low binding energy, which may involve Van der Waals forces, hydrogen bonds, ionic, and hydrophobic interactions. The immobilization method should ensure enzyme stability for long periods, in addition to preventing desorption, making the enzyme reusable and allowing for the free diffusion of substrates and reaction products [57,59,60]. 

**Figure 1:**
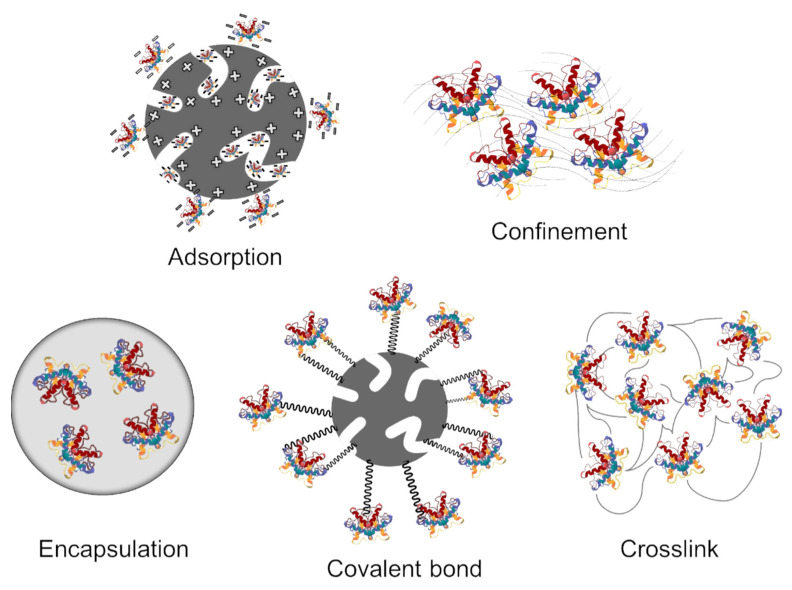
Scheme of the enzyme immobilization methods.

As reported by Alnoch et al. [13], several parameters should be considered when selecting the immobilization method, including the support used, the efficiency of the enzyme, the costs of the immobilization procedure, the toxicity of the immobilization reagents, and the final properties of the immobilized biocatalyst, so that the enzymes can be immobilized by different methods, as can be seen in more detail in Figure 1.

Regarding protease immobilization protocols, they vary depending on the enzyme used, since each enzyme has its optimal range of immobilization pH and temperature. For example: Silva et al. [17] immobilized papain and pancreatin using pH 7.5 and 10 g of support for 90 min of contact at 25 °C. Gu et al. [61], using 10 g of support to immobilize papain (1 mg/mL) at pH 7 for 4 h at room temperature. Huang et al. (2018) immobilized chymotrypsin at pH 6. Santos et al. [10] immobilized pepsin (2 mg/mL) in 100 mg of support at pH 3 at 25 °C for 2 h. Ahmed et al. [62], immobilized caseinase (1247 U) in 0.1 g of support at pH 6 at 4 °C for 24 h. Souza et al. [5] immobilized trypsin (1 mg/mL) in 50 mg of support in medium with pH 8 at 25 °C for 2 h. Santos et al. [63] immobilized pepsin (1 mg/mL) on 50 mg of support in a medium with pH 3 at 25 °C for 2 h. Santos et al. [64] immobilized trypsin (1 mg/mL) in 50 mg of support in a medium with pH 5 at 25 °C for 2 h. Miguez et al. [65] used a 1:20 (m·v^−1^) ratio (support/trypsin solution) at 25 ◦C for 12 h in different pH ranges. In general, it is worth noting that each enzyme and/or support has optimal application conditions, and it is not possible to establish a general immobilization protocol. These conditions are variable according to the structure of the enzyme and the distribution of residual amino acids in the structure, as well as the reactive groups present on the support.

### 3.1. Protease Immobilization

#### 3.1.1. Methods for Protease Immobilization

As reported by Demirkan et al. [66], the immobilization of proteases increases their half-life, facilitates the separation of the enzyme from the reaction products, and allows for their use in conditions that would not be efficient in their native form, in addition to preventing the losses of catalytic activity associated with structural modification or autoproteolysis, and providing resistance to denaturing and microbial attacks.

Although there are several techniques for enzyme immobilization, the most used techniques are adsorption (physical method), cross-linking, and covalent bonding (chemical method). Concerning the immobilization by covalent bonds, glutaraldehyde stands out as the modifying agent, due to its efficiency and versatility, being used to immobilize a series of enzymes on different types of supports [67]. Further studies on modifying agents to improve the properties of proteases immobilized by covalent bonds have been performed.

Metalized matrices have attracted great attention, as they allow for the use of previously known porous matrices, and the insertion of metallic groups leads to the formation of chemical bonds with the enzyme, with promising results when compared to other agents. Proteases immobilized by covalent bonds on metalized supports showed greater binding efficiency, improvements in catalytic efficiency (better exposure of the active sites by avoiding steric hindrance), better chemical/thermal stability, greater resistance to denaturation by changes in pH and temperature, water-miscible and immiscible organic solvents, detergents, and others [68,69].

According to Calzoni et al. [70], the use of immobilized enzymes, mainly proteases, in industrial processes is consolidating. Among the industries that benefit from the hydrolytic properties of proteases, the food, detergent, pharmaceutical and leather industries stand out [71]. Among the applications of immobilized proteases, the main highlight is the production of protein hydrolysates and products of high added value through the degradation of proteins and biomass residues. Wei et al. [72] obtained flaxseed protein hydrolysates using alcalase and flavorzyme, immobilized in calcium alginate. Zhu et al. [73] demonstrated an improvement in soy protein stability and hydrolysis using alkaline proteases in magnetic nanoparticles. Husain [68] highlights the use of alkaline proteases (chymotrypsin, papain, subtilisin and thermolysin) immobilized in nanomaterials being used for the synthesis of new peptides, the hydrolysis of different proteins and the structural analysis of proteins more efficiently than in their soluble form. Soy protein isolate hydrolysate was obtained using alcalase immobilized on chitosan magnetic nanoparticles [74]. Active peptides, obtained from the hydrolysis of corn zein, were produced through the use of alcalase and trypsin co-immobilized in calcium alignate-chitosan beads [75]. Husain [68] also highlights the industrial application of proteases immobilized on nanosupports (nanoparticles, nanofibers, nanotubes, and nanoporous matrices) for the synthesis of aspartame.

#### 3.1.2. Supports for Enzyme Immobilization with Potential for Application in the Immobilization of Proteases

The choice of the appropriate immobilization matrix plays a vital role in enzyme immobilization, as well as in its hydrolytic activity, which can lead to an increase in the production of protein hydrolysates [76]. The selection of support depends on the properties of the material, including mechanical strength, physical and chemical stability, hydrophilic/hydrophobic character, adsorption capacity, and operating costs. Generally, the support should provide two main requirements: (i) have a sufficient amount of functional groups on the surface to interact with the enzyme; and (ii) present mechanical properties and dimensions that allow stable performance and the possibility of repeated use for many cycles or in the application of a continuous process [21,77,78].

There is no universal support material suitable for all enzymes or their applications.

The most common or traditional material supports used for enzyme immobilization can be inorganic materials, such as alumina, silica, porous glass, ceramics, diatomaceous earth, clay, and bentonite, activated carbon or organic materials, such as synthetic polymers (poluaniline, polestyrene, poly (vinyl alcohol) and polypropylene or biopolymer such as cellulose (CMC, DEAE-cellulose), starch, chitosan, soponges, agarose, and nanocrystal [79]. 

The need for the presence of different functional groups (such as –OH, COOH, C=O, –SH, –NH2) on the surface of these support materials is dependent on if they are used for immobilization by adsorption or by covalent bonds between enzyme support. 

The discovery and use of new materials has caused this list to continue to grow. For this reason, supports like magnetic nanoparticles, ceramic particles, carbon nanotubes, and graphene, among others, or combinations of these (hybrids or composite) for enzyme immobilization can offer properties designed for particular enzymes or the requirements of a given technological process [78]. Table 1 presents studies on the different methods for protease immobilization.

As previously highlighted and presented in Table 1, there are several methods and matrices that can be used in enzymatic immobilization, such as chitosans, activated carbons, polymeric resins, and silicas [99]. However, it is worth mentioning that, for industrial application, the use of more economical methods and supports become more advantageous. The methods of physical immobilization (adsorption and entrapment) and chemical immobilization (covalent bonding and cross-linking) are the most used. In this way, supports for enzymatic immobilization, in addition to promoting the interactions necessary for the application of these methods, must present physical and chemical characteristics, such as mechanical resistance, the presence of oxygenated functional groups and thermal resistance, which meet these requirements and, in addition, must present a low cost of synthesis. Vallés et al. [100] used gamma alumina (aluminum oxide) beads support to immobilize plant cysteine protease granulosaín, increasing stability with good catalytic capacity and possibility to reuse. In addition, these enzymes were successfully used to hydrolyze dairy proteins [100].

According to Wongrod et al. [101], activated carbon stands out among other supports due to its high chemical, mechanical, and thermal resistance, and hydrophilicity, in addition to presenting a high BET surface area and defined porosity. Furthermore, the surface of activated carbon can be modified by different methods, to create and/or increase the functional groups on its surface, which vary with the type of activation during the synthesis step. In addition to presenting all these characteristics already mentioned, activated carbons can be obtained from agro-industrial residues, generating a low production cost, and its use as a support for enzyme immobilization can increase the value of the product and the cost–benefit ratio. Bijoy et al. [102] highlights that the use of agro-industrial waste for the development of enzyme immobilization matrices, such as activated carbon, will help with waste management, at the same time as being economically viable, promoting sustainability at several levels.

As shown in Table 1, studies on enzyme immobilization by chemical bonds using modified supports have been more recurrent due to increased immobilization capacity and, consequently, better enzymatic performance. Whereas studies on the immobilization of pepsin and trypsin are not as recurrent as those of other proteases, further studies are required on the immobilization and use of the immobilized enzymes. These enzymes have high hydrolysis specificity, that is, they only hydrolyze some specific amino acid sequences, always generating the same peptides at the end of the hydrolysis. Tavano et al. [1] have stated that the use of these enzymes is quite recurrent for the identification of protein constituents in protein hydrolysates by mass spectrometry due to the formation of short-chain peptides with a basic C-terminal.

### 3.2. Activated Carbon as a Support for Enzyme Immobilization

Activated carbon (AC) is a carbonaceous material with a well-developed porous structure, responsible for providing a high specific surface area (BET). It has on its surface heteroatoms of oxygen, nitrogen, and hydrogen, bonded to carbon atoms. The well-developed porous structure is due to the presence of micropores (pores < 2 nm), mesopores (pores ranging from 2 to 50 nm), and macropores (pores > 50 nm). Due to its textural characteristics, activated carbon can adsorb molecules present in both gas and liquid phases. It is also a material with a high mechanical strength and high chemical stability, considered a non-graphitic material because its carbon atoms are arranged in a two-dimensional hexagonal structure. However, activated carbon is not a truly amorphous material, due to the presence of a microcrystalline structure that differs from the structure of graphite [103].

In the synthesis of activated carbon, compounds rich in carbon can be used, such as bones, sawdust, algae, agro-industrial residues, and lignocellulosic materials, among other carbonaceous materials [104]. Agro-industrial residues, such as bark and pits, have high concentrations of cellulose, lignin, and hemicellulose in their composition, which can be converted into commercial products with high added value, although they are often discarded into the environment. In the last decade, research has focused on the synthesis of activated carbon from various agro-industrial by-products, with special attention to those produced from the reuse of lignocellulosic residues (biomass), thus generating materials with low production costs [103].

These materials undergo activation and carbonization steps to develop internal pores and create surface functional groups. The activation process can be performed by physical, chemical, or physicochemical methods. Regarding the physical activation, the precursor material is thermally treated in a mildly reactive atmosphere, such as water vapor or carbon dioxide, with simultaneous activation and carbonization. In turn, the chemical activation consists of the previous impregnation of the precursor material with chemical agents, such as phosphoric acid (H_3_PO_4_), zinc chloride (ZnCl_2_), potassium hydroxide (KOH) among others, followed by carbonization at high temperatures and in an inert atmosphere [105]. Activated carbon has great industrial versatility and low production cost when compared to other adsorbent materials, which makes it an important and more advantageous alternative for a large class of applications when compared to other porous materials [106].

Activated carbon has been used since 2000 BC when the Egyptians used carbon for water purification. It was later used in granular form during World War I to produce gas masks. After the 1950’s, activated carbon powder was developed and widely used to purify water and control the emission of pollutants [107]. From 1974 onwards, the first industrial application of activated carbon took place in England, as a bleaching agent in the sugar industry, in filters in sewage ventilation systems to eliminate unpleasant odors, and in gas masks to prevent the inhalation of mercury vapors in chemical industries [107]. As activated carbon is inert, it is also used in the purification of chemical compounds, clarification processes, and the removal of flavors and odors from oils, alcoholic beverages, chemical and pharmaceutical products, and wastewater treatment. It is also widely used on an industrial scale as an adsorbent, mainly in the purification/separation of liquids and gases and as catalytic support [108,109]. These applications make activated carbon a product of great interest in diverse areas, such as food, pharmaceutical, chemical, oil, nuclear, automobile, and mining, as well as in the treatment of drinking water, industrial water, and atmospheric air [110,111].

According to Bassan et al. [112], activated carbons are also used as a support in inorganic catalysis and enzyme immobilization due to their properties such as high surface area, good chemical, mechanical and thermal stability, hydrophilicity, and insolubility. Therefore, many studies have been carried out to evaluate the efficiency of activated carbons in different enzyme immobilization processes, as shown in Table 2. These characteristics associated with its low synthetic cost make it highly desirable for enzyme immobilization. Activated carbon also has good physical-chemical resistance due to its electron accepting and donating properties, in addition to the characteristics mentioned above.

As seen in Table 2, different studies were carried out on the immobilization of enzymes in activated carbon, and in these studies the main reasons for using this matrix are its physical and chemical characteristics (high mechanical strength, high chemical stability, resistance to microbial attacks, chemical inertness, biocompatibility, high surface area, defined porosity, presence of surface functional groups, etc.). It is still possible to observe that the derivatives obtained from activated carbon promoted a considerable number of cycles of use, maintaining the activity of the immobilized enzymes. Considering its application on an industrial scale, a support that has a lower synthesis cost and still has the ability to maintain a high number of cycles is desirable, offsetting the costs of the immobilization process. Thus, further studies involving the immobilization of enzymes on activated carbon and their functionalized synthesis are needed.

Immobilization by physical adsorption is the most used method for activated carbon due to its properties, ease of application, and low cost, despite some disadvantages due to the interaction forces, as previously discussed. On the other hand, studies on the modification of the surface of these supports have been performed to improve the enzyme immobilization through the formation of more stable and irreversible bonds, such as covalent bonds. Covalent binding is usually established between amino groups, sulfhydryl groups, and hydroxyl groups of the phenolic ring of the amino acids of the enzyme and the reactive groups of the support [5,103].

According to Zdarta et al. [78], the modifications of supports for enzyme immobilization can improve their catalytic efficiency due to the minimization of the diffusional effects of substrates during the reaction, in addition to improving the stability in continuous and discontinuous processes. Since, when the enzyme is immobilized by adsorption (support without modifications) it is located inside the pores of the carrier, diffusional limitations must also be considered. The transport of substrates and products is restricted as they must be transferred (by convection and/or diffusion) from the solution and diffused to the enzyme catalytic sites. When immobilized on a modified support, the immobilized enzymes are found on the surface of the support, linked by the spacer arms, leaving their catalytic site more available, and facilitating their access to the substrate [10,78]. Consequently, methods of chemical modification of supports have been studied to make them more suitable for use in immobilization by covalent bonding.

#### 3.2.1. Modification of Activated Carbon

The conversion of common support into a heterofunctional support (a heterofunctional support for enzyme immobilization with distinct functionalities, allowing diverse physical and/or chemical interactions with enzymes) through surface modification is effective for enzyme immobilization since the different functional groups formed on the surface of the material can interact with enzymes by chemisorption (covalent bonding) and physisorption (interactions bonds, hydrogen bonds, and interfacial activation), providing the derivatives with high stability, catalytic activity, and selectivity [113,114].

According to Bezerra et al. [51], the increase in the reactive groups on the surface of solid matrices through physical, chemical, and morphological modifications of the supports can produce derivatives with greater catalytic efficiency, leaving its enzyme catalytic site more available, facilitating its access to the substrate, due to the minimization of the diffusional effects of substrates and products during the reaction, in addition to improving the stability in continuous and discontinuous processes, which arouses industrial interest for these biocatalysts [19].

**Figure 2:**
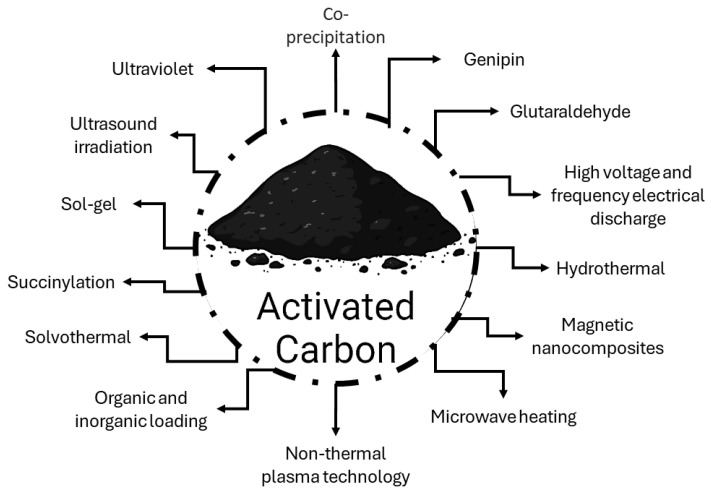
Scheme of possible modifications to activated carbon.

Over the last decade, scientific attention has been directed towards hybrid and composite materials, which combine properties and, therefore, maximize their advantages. Thus, several types of surface modifications have been carried out as an alternative to improve the properties of supports. Modifications of activated carbon consist of changes in its surface through the inclusion of certain procedures or chemical components during activation or after carbon synthesis. These modifications mainly involve chemical changes on the surface and consequently on its properties, with the insertion of several functional groups as the main interest. Modifications of the support can improve the efficiency of the enzyme immobilization, as the support/enzyme interactions are stronger and more stable, leading to improvements in the performance of derivatives [78,115]. Activated carbon can undergo several modifications on its surface, as illustrated in Figure 2.

Among the modification methods on the surface of activated carbon, the glutaraldehyde method stands out, being responsible for forming multi-point covalent bonds between the support and the biomolecules. It is an effective method concerning the thermal and operational stability of enzymes, increasing their rigidity and ensuring greater resistance to small conformational changes caused by temperature variations, organic solvents, and denaturing agents, among others. This stabilization is due to the formation of covalent bonds between aldehyde groups on the support and amine (NH2), thiol (SH), hydroxyl (OH) groups, among other amino acid residues of proteins in enzymes. However, it has some disadvantages, such as the spontaneous polymerization of glutaraldehyde in an aqueous medium, which can lead to a loss of aldehyde groups to interact with enzymes, along with the glutaraldehyde toxicity [10,19].

Although the functionalization with glutaraldehyde is highly efficient for enzyme immobilization, new methods for the surface modification of supports have been studied. Among the substitutes for glutaraldehyde, genipin stands out, a natural compound that has a toxicity around 10,000 times lower than that of glutaraldehyde. Recently, genipin has been used as an activating and/or cross-linking agent for chitosan, gelatin, collagens, among others, for application in several segments, mainly in the area of biotechnology [116]. 

In addition to being a natural compound with low toxicity, genipin spontaneously reacts with primary amines in glucosamines and/or proteins forming covalent bonds, thus increasing the stability of the interactions [117]. Due to its activating and cross-linking properties, it can be used for enzyme immobilization, the encapsulation of compounds, amino acid quantification (it produces blue pigments when in contact with amino acids), emulsion stabilization (O/W), gradual release of drugs in specific regions, constitution of prostheses, among others [118].

Pego et al. [115] studied the modifications of activated carbons using high voltage current and frequency electrical discharge (corona). This method aims to oxidize the surface of the activated carbon through the ionization of gases (O_3_ and O_2_) between the electrode and the substrate through electrical discharges, forming covalent bonds, especially between the C and O atoms, leading to a more reactive surface.

In addition to the modifications after obtaining activated carbon, changes can be performed during the synthesis process through carbonization in autoclaves, a method known as hydrothermal carbonization. This method has stood out due to its simplicity and the possibility of increasing the carbonyl functional groups on the material surface. During the synthesis, oxygen atoms are inserted into the matrix of the precursor material, increasing the acidity of the matrix with an increase in surface polar groups. These new groups on the surface of the adsorbent are responsible for favoring the chemical reactions between the support and the adsorbate, such as the formation of surface complexes, π-cation bonds, electrostatic attraction, and ion exchange [115,119].

Currently, nanomaterials, magnetic materials, and/or magnetic nanocomposites are emerging as new enzymatic immobilization agents. The modification of inert supports with these metallic particles has also been used, as the functional groups formed and the high surface binding energy can increase the amount and stability of the immobilized enzyme [120]. 

Different methods for the surface modification of carbonaceous materials have been studied to improve the enzyme immobilization performance. Among them, modifications using metallization with iron ions have been stood out as one of the most effective methods as the magnetic properties are transferred to the support after impregnation. Modification using iron salts (Fe_3_O_4_; (NH_4_)_2_Fe (SO_4_)_2_; Fe (NO_3_); FeCl_3_; FeSO_4_) can be performed during the activation process or after carbonization. Both modifications are intended to form magnetite (Fe_3_O_4_), due to its predisposition to exhibit magnetic properties, low cytotoxicity, good biocompatibility, and stability in a variety of physiological conditions [120,121,122].

The enzyme immobilization by metal ions is known as the IMAC technique, and it is based on the differential affinity between the bivalent metal ions (Ni^2+^, Zn^2+^, Cu^2+^, Ca^2+^, Co^2+^, or Fe^2+^) supported in a solid matrix by exposed groups of enzymes. The affinity is due to reversible coordination bonds formed between a chelated metal ion (the adsorption center) and certain amino acid residues, such as histidine imidazole, cysteine-thiol, and tryptophan indole, which donate electrons to the metal ion, acting as a Lewis base [88,123]. According to Ding et al. [124], immobilization using bivalent metal ion modification has many advantages, including an improvement of the immobilization capacity and enzyme activity, in some cases.

Table 3 presents some studies on the modification of activated carbon and its application for enzyme immobilization. It is worth mentioning that there are no studies with activated carbon used as the support for protease immobilization. Therefore, a more in-depth investigation of the application of this matrix and its modifications to improve the enzyme immobilization process is required.

As can be seen in Table 3, different studies on the surface modifications of activated carbons have been conducted in recent years, proving that this support has great versatility, and its surface can be modified by different methods. The physical properties of activated carbon, which give it a rigid structure, make it resistant to the stages of modification, undergoing little or no change in its morphology. There are no reports in the literature about support disintegration during the modification or reuse steps. In addition, its porous structure, high surface area and the presence of surface functional groups ensure the fixation of new functional groups capable of improving the immobilization capacity and activity of immobilized enzymes.

#### 3.2.2. Proteases Immobilized on Activated Carbon

Even though there are few studies describing the use of proteases in combination with activated carbon, it has stood out as a support for the immobilization of this class of enzymes due to its unique characteristics. Llorente et al. [126] used activated carbon to immobilize proteinases extracted from artichoke (*Cynara scolymus* L.) and used them for milk coagulation and adsorption, with activated carbon leading to the isolation of a heterodimeric milk-clotting proteinase consisting of subunits of 30 and 15 kDa subunits. Capobiango et al. [127] immobilized pancreatin on activated carbon to remove the phenylalanine (Phe) present in corn proteins, highlighting that the use of activated carbon is advantageous for Phe removal. Ganesh Kumar et al. [128] used highly porous activated carbon as a support for the immobilization of acidic protease, which maintained around 50% of its initial activity, while the free enzyme was completely inactivated.

Kumar et al. [129] used functionalized mesoporous activated carbon as a support material for immobilizing the acid protease to obtain the catalytic efficiency of the enzyme for more than five consecutive reaction cycles. Kumar et al. [130] used mesoporous activated carbon for in situ immobilization of acidic protease to maintain significant catalytic efficiency for more than ten consecutive reaction cycles. Salleh demonstrated that activated carbon obtained from agro-industrial waste (rice husk) showed a high immobilization efficiency of a neutral protease.

Peres et al. [131] demonstrated that activated carbon has a high capacity to immobilize papain. Salleh et al. [132] demonstrated that proteases immobilized in activated carbon were stable when subjected to adverse conditions: solvent systems (polar and nonpolar), organic matter (lipids), metal ions, and surfactants. Pousamy et al. [133] studying the immobilization of halotolerant protease, obtained from *Lysinibacillus macroides*, immobilized on activated carbon functionalized with glutaraldehyde, reported that it was possible to immobilize the protease and that it efficiently degrades proteins in tannery wastewater. Santos et al. [63] evaluated the different types of modifications in activated carbon (functionalization with glutaraldehyde, genipin, and metallization in the presence and absence of chelating agent) against the efficiency of pepsin immobilization. They observed that functionalization using iron ions allowed an immobilization capacity greater than 99%, and enzymatic activity close to that of the enzyme in the native form. 

Xiong et al. [134] immobilized alkaline protease on activated carbon for the production of high Fischer ratio oligopeptides. Santos et al. [64], immobilized trypsin on activated carbon subjected to different modifications (glutaraldehyde, genipin, and metallization) and demonstrated that trypsin immobilized on activated carbon modified with iron ions in the presence of a chelating agent presented better results in hydrolytic activity, in addition to presenting better operational stability when comparing with the soluble enzyme. 

**Figure 3:**
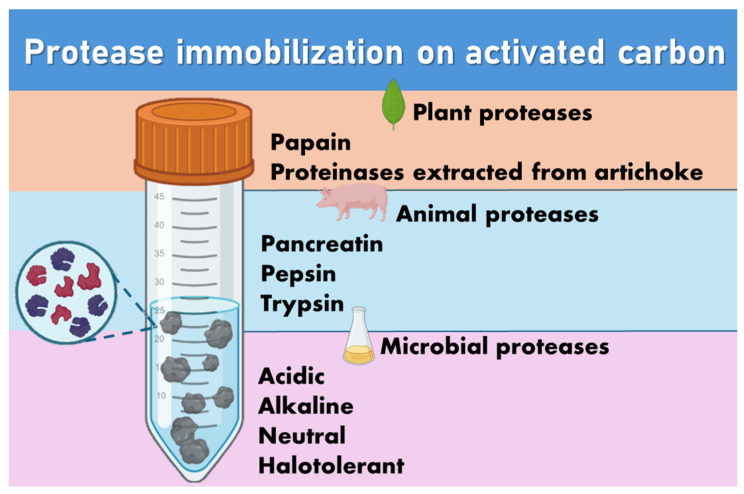
Proteases immobilized on activated carbon.

It can, therefore, be observed that over the years, research seeking new modifications to activated carbon so that it increases the activity of immobilized proteases has been intensified to improve the application of this class of enzymes that have great industrial versatility. Proteases that have already been immobilized on activated carbon can be seen in Figure 3.

## 4. Immobilized Protease Application

As we know, proteases have a very short half-life, so immobilization is necessary to obtain a stable form of the enzyme to expand and facilitate its application in various industrial sectors. According to Maghraby et al. [135], immobilized enzymes can be applied in several industrial sectors: detergent, textile, pharmaceutical, medical, food industries, water treatment, and sewage/effluent recycling, among others. Morellon-Sterling et al. [136], reported several studies regarding the application of immobilized pepsin in different ways (in porous glass spheres, in a continuous reactor, and a fluidized bed reactor) to obtain milk coagulation. Luk et al. [137] emphasize the use of pepsin immobilized in porous chitosan spheres for protein hydrolysis to extract peptides by continuous hydrolysis. Motoi et al. [138] highlighted the use of pepsin and trypsin immobilized in agarose hydrogels for use in mass spectrometry. In the food industry we can highlight the application of an alkaline protease immobilized on a mesoporous support (zeolite/silica) used in the conversion of milk into cheese. Immobilization guaranteed the preservation of activity by 74% after 16 days of storage when compared to free protease, which preserved only 50% of the initial activity [139]. In addition, Benucci et al. [140] immobilized protease (endopeptidase) obtained from *Aspergillus niger* by cross-linking in chitosan to eliminate gluten from beer, resulting in an 80% reduction in gluten content after 10 h of treatment.

## 5. Final Considerations

Enzymes are biocatalysts with several applications in different segments, mainly in the food industry. Proteases stand out among the classes of enzyme due to their greater versatility. However, their use on an industrial scale is associated with high costs and low stability under adverse conditions, thus they are considered barriers to operations on a large scale.

Therefore, several studies on enzyme immobilization have been carried out to prevent the problems associated with the use of native enzymes. The immobilization technique consists of imprisoning or binding the enzyme to support, so that the enzymes continue to show catalytic activity, with the possibility of reuse of these biocatalysts. There is a growing demand for applications of immobilized enzymes, thus the synthesis of new supports, as well as the modification of supports to improve the immobilization efficiency without a loss of enzymatic activity, is of paramount importance. Inorganic supports stand out in this process due to their low synthesis cost, inert profile, as well as their high mechanical strength, good thermal stability, and resistance to organic solvents and attack by microorganisms. 

Among the supports, activated carbon has stood out as a matrix for enzyme immobilization due to its physical properties, such as a highly developed porous structure and high BET surface area, combined with its surface composed of heteroatoms, which can improve immobilization by adsorption. Activated carbons and other inorganic and organic supports are susceptible to modification, being classified as heterofunctional supports due to the insertion of reactive groups. These groups allow for the formation of more versatile interactions since they can interact with enzymes by chemisorption and physisorption, providing better stability on the support.

In general, there are few studies on protease immobilization, mainly on activated carbon, thus a more in-depth study is required on this matrix susceptible to several surface modifications. Studies on the modifications with insertion of metal ions and/or oxygenated functional groups are needed, once these groups allow better interaction between the enzyme and the support, leading to better enzyme immobilization and activity.

## Figures and Tables

**Table 1:** Protease immobilization using different supports and immobilization methods.

Support	Enzyme	Method	Results	References
**Physical (Adsorption)**				
**Celite**	Ficin	pH 7 for 10 min	Immobilized enzyme activity of 160 U/mg for casein hydrolysis	[80]
**Activated carbon**	Papain	pH 7.5 for 0.5 h	Immobilization capacity of 97 mg/g and enzyme activity of 75 mg Phe/100 g for whey hydrolysis	[17]
**Activated carbon**	Pancreatin	30 min; 25 °C	Immobilized enzymes with 84% activity for the removal of phenylalanine	[17]
**Polymer-modified chitosan/clay Composite**	Papain	pH 7; 20 °C	Immobilization capacity of 34.47 mg/g and residual catalytic activity of 100% for BAEE hydrolysis	[81]
**Multi walled carbon nanotubes**	Papain	pH 7; 2 h; 200 rpm	Immobilization efficiency of 4.2 mg/mL with enzyme activity of 67% for casein hydrolysis	[82]
**Magnetic Chitin Nanofiber Composite**	α-chymotrypsin	2 h; 20 °C; 200 rpm	Immobilization capacity of 92.4 mg/g with a relative activity of 100% for casein hydrolysis	[83]
**Chitin**	Proteases	pH 7.5; 4 °C overnight	Recovered enzyme activity of 2.5% for casein hydrolysis	[84]
**Activated carbon**	Pepsin	pH 3; 2 h, 30 rpm	Immobilization efficiency of 93.6% with enzyme activity of 1.3 U·mg^−1^ for hydrolysis of bovine casein	[10]
**Activated carbon**	Trypsin	pH 8; 2 h, 30 rpm	Immobilization efficiency of 87.5% with enzyme activity of 2.5 U·mg ^−1^ for hydrolysis of goat casein	[5]
**Activated carbon**	Trypsin	pH 5, 30 rpm, 2 h	Immobilization efficiency of over 91% with enzymatic activity of 2.60 U for casein hydrolysis	[64]
**Chitosan**	Trypsin	pH 9, 200 rpm, 12 h	Immobilization efficiency of over 19% with enzymatic activity of 21.1 nmol·min^−1^·mg^−1^ for BSA hydrolysis	[65]
**Activated carbon**	Pepsin	pH 3, 30 rpm, 2 h	Immobilization efficiency of 98% with enzymatic activity of 0.95 U for casein hydrolysis	[85]
**Chemical (Covalent bonding)**				
**Glutaraldehyde-activated silica**	Trypsin	pH 7.5, 4 °C, 1 h, 200 rpm	Immobilization efficiency of 63% with enzyme activity of 92 nmol/min/mg for BSA hydrolysis	[86]
**Silica-coated Fe_3_O_4_ nanoparticles**	Papain	pH 7.5, 2 h	Immobilization efficiency of 57.9% with enzyme activity of 86% for hydrolysis of bovine casein	[87]
**Carbon coated nanoparticles**	α-chymotrypsin	-	Immobilization capacity of 50 mg/g with 25% hydrolysis activity of N-benzoyl-L-tyrosine ethyl ester substrate	[88]
**Magnetic chitosan nanoparticles**	Pepsin	-	Immobilization capacity of 99 mg/g with enzyme activity of 85% for amide hydrolysis	[89]
**Poly (ethylene terephthalate) (PET) with PVA**	Trypsin	pH 5.5, 2 h	Immobilization capacity of 0.62 µmol pNA min^−1^g^−1^ mat for BAPNA hydrolysis	[90]
**Glutaraldehyde-modified chitosan**	Papain	pH 8, 5 h	Enzyme activity of 2.7 U/g for hydrolysis of azocasein sulfanilamide	[91]
**Glutaraldehyde-modified chitosan**	Stem Bromelin	pH 3.2, 150 rpm, 20 °C overnight	Immobilization efficiency of 41%	[92]
**Pineapple Peel Carboxymethyl Cellulose (PCMC)/Polyvinyl Alcohol (PVA)/Mesoporous Silica SBA-15 hydrogel composites**	Papain	pH 6.5, 1.5 h	Immobilization capacity of the hydrogel of 100% with enzyme activity of 1800 U/g for casein hydrolysis	[93]
**Glyoxyl-agarose support**	Ficin	pH 10; 25 °C, 3 h	Immobilization efficiency of 100% and relative activity of 40% for the hydrolysis of Benzoyl-arginine-p-nitroanilide (BANA)	[67]
**Magnetic chitosan nanoparticles**	Trypsin	pH 7.5, 25 °C, 1 h, 200 rpm	Immobilization capacity of 149.25 mg/g with residual activity of 100% for BAEE hydrolysis	[94]
**Magnetic Chitin Nanofiber Composite**	α-chymotrypsin	20 °C, 2 h, 200 rpm	Immobilization capacity of 581.84 mg/g with a relative activity of 100% for casein hydrolysis	[83]
**Electrospun PVA Nanofibers**	Ficin	pH 8, 1 h	Immobilization capacity of 92% for hydrolysis of Nα-benzoyl-L-arginine 4-nitroanilide hydrochloride (BAPA)	[95]
**Porous magnetic nanoparticles**	Papain	25 °C, 12 h.	Immobilization efficiency of 82% with a casein hydrolysis capacity of 4.95 mg/L·min	[96]
**Glutaraldehyde-activated agarose beads**	Ficin	pH 7, 25 °C, 4 h	Immobilization efficiency of 100% with enzyme activity of 40% for casein hydrolysis	[97]
**Glutaraldehyde-Modified Chitin**	Protease from sunflower seeds	pH 7.5, 4 °C, 12 h	Recovered enzyme activity of 38% for casein hydrolysis	[84]
**Glutaraldehyde-Modified Chitin**	Trypsin	pH 8.5, 25 °C, 30 min	Relative activity of 100% for hydrolysis of Nα-benzoyl-L-arginine 4-nitroanilide hydrochloride (L-BAPA)	[98]
**Glutaraldehyde-Modified Activated carbon**	Pepsin	pH 3, 30 rpm, 2 h	Immobilization efficiency of 94.9% with enzyme activity of 1.75 U·mg ^−1^ for the hydrolysis of bovine casein	[10]
**Glutaraldehyde-Modified Activated carbon**	Trypsin	pH 8, 30 rpm, 2 h	Immobilization efficiency of 91% with enzyme activity of 3 U·mg ^−1^ for goat casein hydrolysis	[5]
**Activated carbon modified with metal ions**	Trypsin	pH 5, 30 rpm, 2 h	Immobilization efficiency of over 95% with enzymatic activity of 4.11 U for casein hydrolysis	[64]
**Glutaraldehyde–glycine activated chitosan**	Trypsin	pH 9, 200 rpm, 12 h	Immobilization efficiency of over 81% with enzymatic activity of 33.1 nmol·min^−1^·mg^−1^ for BSA hydrolysis	[65]
**Activated carbon modified with genipin**	Pepsin	pH 3, 30 rpm, 2 h	Immobilization efficiency of 98% with enzymatic activity of 1.39 U for casein hydrolysis	[85]

Source: The author, 2024.

**Table 2:** Studies performed using activated carbon as a support for enzyme immobilization.

Method	Application	Activity Free Enzyme	Activity Immobilized Enzyme	References
**Pepsin**				
**Adsorption**	Hydrolysis of bovine casein	41.67 U	245.02 U—8 cycles	[10]
**Covalent bonding**			299.79 U—8 cycles	
**Adsorption**	Hydrolysis of bovine casein	3.32 U	1.04 U—1 cycle	[63]
**Covalent bonding (glutaraldehyde)**			1.10 U—1 cycle	
**Covalent bonding (genipin)**			1.84 U—1 cycle	
**Covalent bonding (metal ions)**			2.30 U—1 cycle	
**Adsorption**	Hydrolysis of goat casein	2.90 U	4.35 U—8 cycles	[85]
**Covalent bonding (glutaraldehyde)**			3.50 U—8 cycles	
**Covalent bonding (genipin)**			6.35 U—8 cycles	
**Trypsin**				
**Adsorption**	Hydrolysis of goat casein, among others	3.35 U	9.22 U—4 cycles	[5]
**Covalent bonding**			10.45 U—4 cycles	
**Adsorption**	Hydrolysis of bovine casein	3.76 U	3.30 U—2 cycles	[64]
**Covalent bonding (glutaraldehyde)**			3.20 U—2 cycles	
**Covalent bonding (genipin)**			5.45 U—4 cycles	
**Covalent bonding (metal ions)**			16.74 U—6 cycles	

Source: The author, 2024.

**Table 3:** Modification of carbonaceous supports for enzyme immobilization.

Activated Carbon	Modification	Enzyme	References
**Commercial**	Copper Phosphate Magnetization	Papain	[125]
**From pupunha palm**	Modification with glutaraldehyde	Pepsin	[10]
**From yellow mombin fruit stones**	Modification with glutaraldehyde	Trypsin	[5]
**From tamarind seeds**	Modification with: Glutaraldehyde; Genipin; iron salts (Fe^2+^ and Fe^3+^)	Pepsin	[63]
**From tamarind seeds**	Modification with: Glutaraldehyde; Genipin; iron salts (Fe^2+^ and Fe^3+^)	Trypsin	[64]
**From Umbu seeds**	Modification with: Glutaraldehyde; Genipin	Pepsin	[85]

Source: The author, 2024.

## Data Availability

Not applicable.
